# Identification of Feldin, an Antifungal Polyyne from the Beefsteak Fungus *Fistulina hepatica*

**DOI:** 10.3390/biom10111502

**Published:** 2020-10-31

**Authors:** Jungho Lee, Yi-Ming Shi, Peter Grün, Matthias Gube, Michael Feldbrügge, Helge Bode, Florian Hennicke

**Affiliations:** 1 Institute for Microbiology, Cluster of Excellence on Plant Sciences, Bioeconomy Science Centre, Heinrich Heine University Düsseldorf, 40204 Düsseldorf, Germany; jungho.lee@hhu.de (J.L.); feldbrue@hhu.de (M.F.); 2 Molecular Biotechnology, Department of Biosciences, Goethe University Frankfurt, 60438 Frankfurt am Main, Germany; Shi@bio.uni-frankfurt.de (Y.-M.S.); p.gruen@bio.uni-frankfurt.de (P.G.); h.bode@bio.uni-frankfurt.de (H.B.); 3 Soil Science of Temperate Ecosystems, Georg-August University Göttingen, 37077 Göttingen, Germany; mgube@uni-goettingen.de; 4 Buchmann Institute for Life Sciences (BMLS), Goethe University Frankfurt, 60438 Frankfurt am Main, Germany; 5 Senckenberg Gesellschaft für Naturforschung, 60325 Frankfurt, Germany; 6 Project Group Genetics and Genomics of Fungi, Chair Evolution of Plants and Fungi, Ruhr-University Bochum (RUB), Universitätsstr. 150, 44780 Bochum, Germany

**Keywords:** agaricomycetes, antifungals, biologicals, polyines, polyynes, polyacetylenes

## Abstract

Fruiting body-forming members of the Basidiomycota maintain their ecological fitness against various antagonists like ascomycetous mycoparasites. To achieve that, they produce myriads of bioactive compounds, some of which are now being used as agrochemicals or pharmaceutical lead structures. Here, we screened ethyl acetate crude extracts from cultures of thirty-five mushroom species for antifungal bioactivity, for their effect on the ascomycete *Saccharomyces cerevisiae* and the basidiomycete *Ustilago maydis*. One extract that inhibited the growth of *S. cerevisiae* much stronger than that of *U. maydis* was further analyzed. For bioactive compound identification, we performed bioactivity-guided HPLC/MS fractionation. Fractions showing inhibition against *S. cerevisiae* but reduced activity against *U. maydis* were further analyzed. NMR-based structure elucidation from one such fraction revealed the polyyne we named feldin, which displays prominent antifungal bioactivity. Future studies with additional mushroom-derived eukaryotic toxic compounds or antifungals will show whether *U. maydis* could be used as a suitable host to shortcut an otherwise laborious production of such mushroom compounds, as could recently be shown for heterologous sesquiterpene production in *U. maydis*.

## 1. Introduction

Many species of Basidiomycota, the second-largest division of the kingdom Fungi, with currently more than 30,000 described species [1], form conspicuous fruiting bodies (basidiomes, basidiocarps, mushrooms) for reproduction. As a result, these fungi are commonly referred to as ‘(basidiomycete) mushrooms’. In particular, fruiting bodies are produced by species of the class Agaricomycetes, which is assigned to the Basidiomycota subphylum Agaricomycotina [2,3]. Especially this subphylum and, in particular, basidiomes of respective species, have been tapped for secondary metabolites. Accordingly, 80–85% of medicinal mushroom products are derived from basidiomes, while only 15% are obtained from mycelia [2]. Like other fungi, mushrooms maintain their ecological fitness mainly by evolving efficient chemical defense mechanisms to protect their vegetative mycelia and their fruiting bodies against antagonists [4]. Hence, they produce a cornucopia of diverse unique bioactive substances. A few such ‘biologicals’ have made it into development as agrochemical pesticides or pharmaceutical lead structures, e.g., the cytotoxic illudins, the antifungal strobilurins, and the antibacterial pleuromutilins [2,5,6]. To date, the biosynthesis of at least two Agaricomycotina molecules has been accomplished by means of heterologous production in an ascomycetous host, including the tricyclic diterpenoid pleuromutilin and the polyketide strobilurin A [7,8]. However, customary hosts, such as the Gram-negative bacterium *Escherichia coli* or ascomycete model fungi like *Saccharomyces cerevisiae,* do not always work well in the expression of many of the numerous relevant metabolite families. Amongst other problems, e.g., on the level of transcription or translation, intermediates or final products biosynthesized via heterologous gene expression can be toxic, and enzymatic activities of heterologously expressed cytochrome P450 monooxygenases may be very low [9,10,11,12]. As an example for final product toxicity, certain antifungals may be more toxic to ascomycetes than to basidiomycetes, such as described by Schlingmann et al. [13]. In that study, the human-pathogenic ascomycetous yeast *Candida albicans* [14] proved more susceptible to the compound of interest than the tested basidiomycetes [13].

The beefsteak fungus *Fistulina hepatica*, formerly a Schizophyllaceae [15], is an edible mushroom *incertae sedis* from the order of Agaricales [1] that is a producer of a number of different metabolites with interesting chemical properties or bioactivities. This wood-decaying species has been genome-sequenced to understand the brown-rot (heart rot) it causes in *Quercus* spp. and *Castanea* spp. [16,17,18]. Among several other metabolites, some bioactive polyynes (‘polyacetylenes’) from *F. hepatica* have been described [19,20,21,22,23,24]. In addition to the above-mentioned fungal biologicals, polyynes comprise another important class of fungal compounds. They are even better known in plants, exemplified by the antifungals falcarinol and falcarindiol from carrot roots [25,26,27].

In the present study, we screened ethyl acetate crude extracts from mycelial cultures of 41 strains representing 35 species of basidiomycete mushrooms against the ascomycete *S. cerevisiae* and the basidiomycete *Ustilago maydis,* identifying one extract from a wild strain of *F. hepatica* that showed selective antifungal activity. Bioactivity-guided substance purification, and, eventually, structure elucidation from one bioactive fraction revealed an antifungal polyyne that we named feldin.

## 2. Materials and Methods

### 2.1. Fungal Material, Fungal Cultivation, and Media

Fruiting bodies of 35 different saprotrophic basidiomycete mushroom species were collected to initiate axenic cultures (Table 1). Their generation, maintenance, and storage is exemplarily described with the fungus that served for production of basidiomycete ethyl acetate crude extract BE05, *F. hepatica*. Basidiomes of *F. hepatica* were collected on August 18th 2013 from an oak tree stump in a deciduous forest on chalky soil dominated by *Fagus sylvatica* and *Quercus* spp. This forest is situated to the East of the German city of Jena, about 300 m to the northeast from the Fürstenbrunnen, the spring of the creek Pennickenbach, and about 1 km to the southwest from the stony monument ‘Steinkreuz Ziegenhain’ (50°54′51.71″ N, 11°38′23.36″ E). Dikaryotic mycelium of *F. hepatica* was isolated by sterile explanting of hyphal tufts onto potato dextrose agar (PDA, 413758.1210, AppliChem, Darmstadt, Germany), complemented with 100 µg/mL ampicillin and 50 µg/mL chloramphenicol from freshly harvested *F. hepatica* fruiting bodies, which were torn open aseptically. Cultures were generally grown at 25 °C in the dark. When hyphal outgrowth from the fruiting body explants occurred after about a week, subcultures were made by transferring hyphal tufts that were taken from the edge of uncontaminated areas. Subcultures were then grown for one week. After one additional round of contamination-free subcultivation, culture characteristics of the pure culture of *F. hepatica* were assessed under the microscope. Cultures were maintained on PDA, and stored as fully grown refrigerated mineral oil stocks [28] at the Department of Mycology (Goethe University Frankfurt, Frankfurt, Germany), using light paraffin oil (J217-500ML, VWR, Radnor, PA, USA) and corn meal agar slants (42347-500G-F, Sigma-Aldrich Chemie GmbH, Munich, Germany).

### 2.2. Microscopy

To confirm the morphological identification, microscopy of the mycelium culture on PDA at 25 °C for 4 weeks was carried out as previously described [29,30], using a wide-field microscope set-up from Visitron Systems (Puchheim, Germany), Axio Imager M1 equipped with a Spot Pursuit CCD camera (Diagnostic Instruments, Sterling Heights, MI, USA), and the objective lens Plan Neofluar (40 ×, NA 1.3; 63 ×, NA 1.25; Carl Zeiss, Jena, Germany). The microscopic system was controlled by MetaMorph software (Molecular Devices, version 7, Sunnyvale, CA, USA). The program was also used for image processing, including the adjustment of brightness and contrast.

### 2.3. Extraction

For each of the 41 basidiomycete strains (see Table 1), two PDA plates (equals about 50 mL volume) grown for four weeks were extracted in their entirety with the twofold volume of pure ethyl acetate (100 mL). For bioactive compound isolation, this step was repeated on a large scale for *F. hepatica*. The plates were sliced and soaked in the twofold volume of pure ethyl acetate shaking overnight at 150 rpm. The extraction procedure was performed twice and the ethyl acetate extract was filtrated through filter paper (Munktell & Filtrak GmbH, Bärenstein, Germany). Ethyl acetate was then evaporated at 40 °C and 180 mbar using a rotary evaporator equipped with a cooling system working at 4 °C. Further removal of residual organic solvent in vacuum yielded basidiomycete ethyl acetate crude extract of each strain from Table 1. In the case of *F. hepatica*, about 3 g of brown ethyl acetate crude extract was obtained from 4000 fully colonized PDA plates.

### 2.4. Liquid Chromatography/Mass Spectrometry

Fractionation and subfractionation of the crude extract and isolation of pure substance were carried out on preparative and semipreparative Agilent LC-MS 1260 Infinity II coupled to a DAD and a single quadrupole detector. The crude extract was resuspended in methanol and then subjected to the preparative HPLC with a C18 column (30 × 250 mm, 10 µm) using an acetonitrile/water gradient (0.1% formic acid) 0–18 min, 5–100%, 40 mL/min to afford eight fractions. Fraction 8.2 (29.5 mg), containing the bioactive compound, was subjected to semipreparative HPLC with a phenyl column (9.8 × 250 mm, 5 µm) using an acetonitrile/water gradient (0.1% formic acid) 0–10 min, 45–60%, 3 mL/min to afford six subfractions. Subfraction 8.2.6 (5.8 mg), mainly containing the target compound, was further purified by the semipreparative HPLC with a C18 column (9.8 × 250 mm, 5 µm) using 45% acetonitrile/water isocratic elution (0.1% formic acid), 3 mL/min to afford feldin (2.0 mg), from which, beforehand, a small subsample (0.3 mg) had been saved and dissolved in DMSO for an immediate bioactivity test against *S. cerevisiae* before NMR. The rest of the 2.0 mg feldin retrieved after NMR were used for another round of bioactivity testing against *S. cerevisiae*.

### 2.5. NMR Spectroscopy

The 1D and 2D NMR spectra were recorded on a 500 MHz NMR spectrometer for ^1^H, and 125 MHz for ^13^C. Chemical shifts (*δ*) were given on parts per million (ppm) scale and referenced to the solvent signals. Coupling constants were expressed in hertz (Hz).

### 2.6. Antifungal and Antibacterial Assays

The antifungal and antibacterial assays were performed using the agar diffusion (Kirby-Bauer) method applying a protocol described previously [31]. *Ustilago maydis* AB33 [32] and *S. cerevisiae* ESM356-1 strains [33] were precultured in CM medium supplemented with 10 g/L glucose [34,35] and YPD, respectively. Cultivation of fungi was performed at 28 °C, shaking in baffled flasks at 200 rpm. The Gram-positive bacterium *Corynebacterium glutamicum* ATCC13032 [36] and the Gram-negative bacterium *Escherichia coli* K-12 derivate Top10 (Life Technologies, Carlsbad, CA, USA) were precultured in LB medium by shaking in baffled flasks at 200 rpm at 28 °C and 37 °C, respectively. Then, 500 μL of diluted overnight cultures to an OD_600_ of 0.5 was inoculated on LB agar plates. Sterile 5 mm diameter Whatman filter paper disks (GE Healthcare Life Sciences, Munich, Germany) were placed on the agar plates. For the antifungal assay, each dried fungal extract, a subfraction of the dried fungal extract or pure feldin compound (100 μg) was dissolved in DMSO to impregnate the disks. In the case of the antibacterial assay, different amounts (100 μg, 200 μg, and 500 μg) of dried basidiomycete ethyl acetate crude extract BE05 were dissolved in DMSO to impregnate the disks. For this, 200 µg nourseothricin (clonNAT, AB-102L, Jena Bioscience, Jena, Germany) was used as the positive control, while DMSO was used as negative control. To observe antifungal activity, inoculated agar plates were incubated at 28 °C for 48 h. In the case of the antibacterial activity assay, the inoculated agar plates were incubated at 28 °C (*C. glutamicum*) or 37 °C (*E. coli*) for 48 h. Afterwards, the growth inhibition zone surrounding the disk was recorded photographically. Three independent biological experiments (n = 3) were carried out.

## 3. Results

### 3.1. Bioactivity Tests with Ethyl Acetate Extracts from 41 Basidiomycete Mushroom Strains

**Figure 1:**
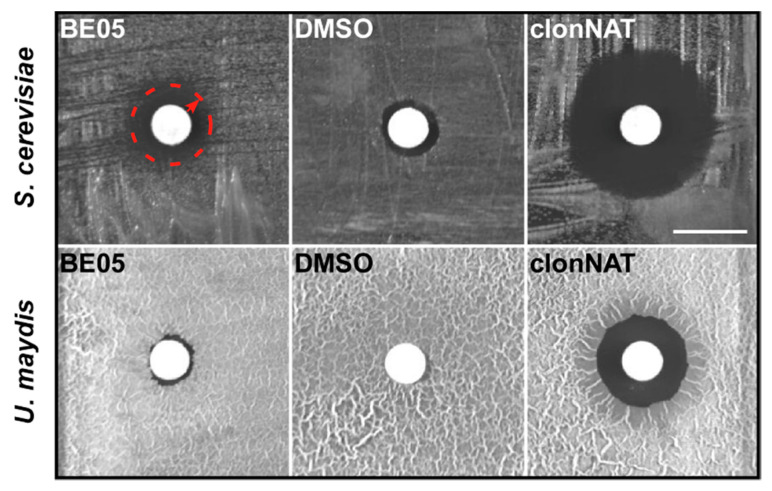
Bioactivity test of the basidiomycete ethyl acetate crude extract BE05 against *S. cerevisiae* and *U. maydis*. In total, 100 µg of crude extract was dissolved in DMSO to impregnate a filter paper disk centrally placed on each agar plate. DMSO was used instead of the crude extract as negative control and 200 µg of clonNAT dissolved in ddH_2_O was used as positive control. The scale bar represents 1 cm. Three independent biological experiments (n = 3) were carried out. The biotesting with extract BE05 (left part of the panel) yielded a zone of growth inhibition (halo, dashed red circle with arrow) with *S. cerevisiae,* while resulting in minimal growth inhibition with *U. maydis*.

In order to identify useful bioactive secondary metabolites, ethyl acetate extracts from mycelial cultures of wild strains of 41 basidiomycete mushroom strains representing 35 species were tested (Table 1). Their antifungal activity was analyzed in an antimicrobial assay against the yeast *S. cerevisiae* and the yeast form of *U. maydis*. Forty extracts showed no activity under these conditions (Figure S1). However, we detected bioactivity of the crude extract BE05 from *F. hepatica* against *S. cerevisiae*. The same extract was hardly active against *U. maydis* (Figure 1).

The antifungal crude extract BE05 was further tested against Gram-positive *C. glutamicum* and Gram-negative *E. coli* bacteria. With both bacteria, application of 500 µg of BE05 extract resulted in the formation of a two-zone halo, the inner zone of which completely lacked bacterial growth. In the adjacent outer halo zone that appeared optically brighter than the inner halo, bacterial lawn was still formed but at a lower density compared to the surrounding biofilm (Figure S2). Compared to *S. cerevisiae* (see Figure 1), where halo formation was already striking with 100 µg of BE05 extract, *C. glutamicum* as well as *E. coli* exhibited only a slight growth inhibition when exposed to BE05 extract.

### 3.2. Characteristics of Fistulina hepatica

**Figure 2:**
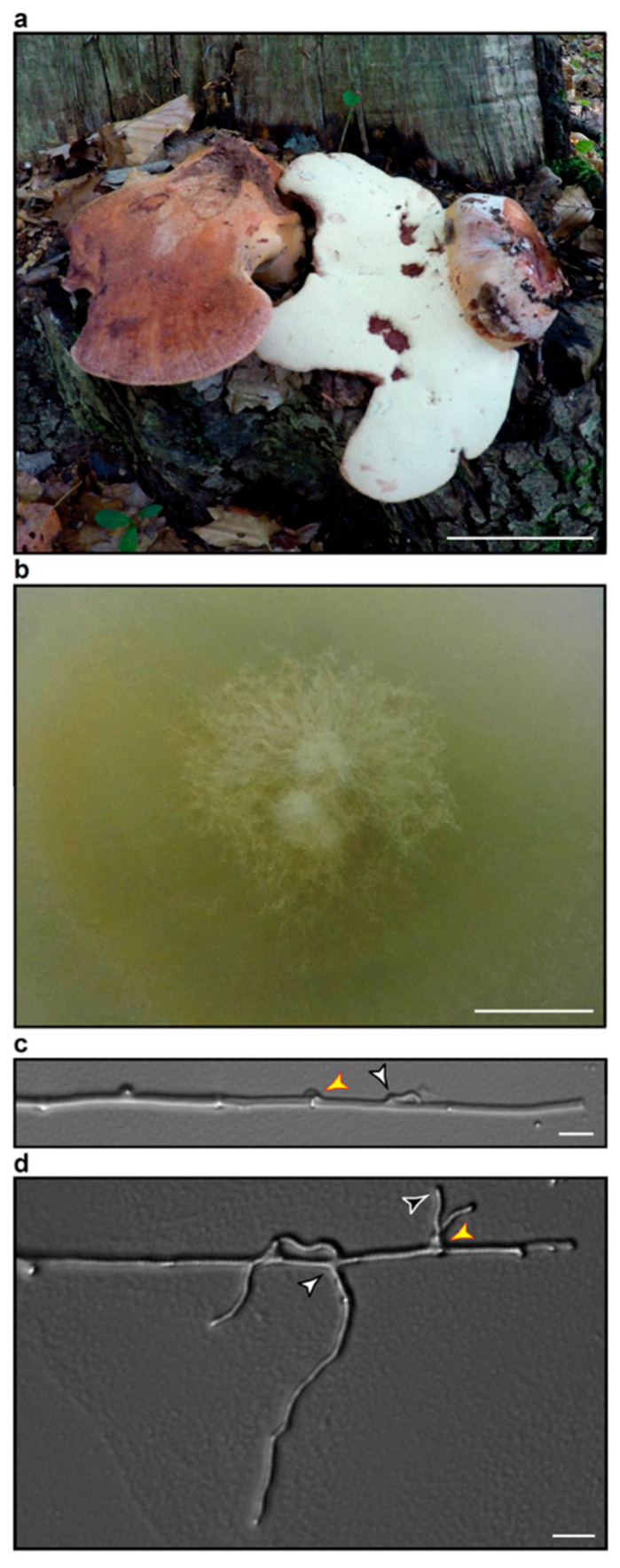
Basidiome and mycelial morphology of the *F. hepatica* wild strain employed in the present study. The shown mycelial culture served to generate basidiomycete ethyl acetate crude extract BE05. (**a**) Pileal surface (left specimen) as well as stipe and pore surface (right specimen) of two fruiting bodies growing from a stump of an oak tree in a mixed *Fagus sylvatica* forest. The scale bar represents 5 cm. (**b**) Mycelial colony morphology of *F. hepatica* growing on potato dextrose agar (PDA) at 25 °C. The scale bar represents 1 cm. (**c**,**d**) Typical hyphal morphology of *F. hepatica,* indicating a clamp connection (red-framed yellow arrowhead) at a septum between two dikaryotic hyphal segments of aerial mycelium at the colony margin. Such clamp connections may grow out to form new hyphae (d, white-framed black arrowhead). Side-branch formation on *F. hepatica* hyphae normally occurs via outgrowth of side branches at acute angles (c-d, black-framed white arrowheads). Such a side branch is either more or less equally sized to the hypha from which it grows out (d, black-framed white arrowhead) or it branches off as a very narrow hypha (c, black-framed white arrowhead) from a wide hypha. The scale bar represents 10 µm.

The fungal strain from which the basidiomycete extract BE05 originates has been isolated from basidiomes of *F. hepatica,* exhibiting unambiguous ecological and morphological features of this species as described by Knudsen and Vesterholt [37], as well as by Krieglsteiner [38]: tongue-shaped basidiome; upper surface reddish-brown; hymenophore light yellowish, consisting of individual tubules; context very soft, reddish-brown, with lighter streaks, exuding a dull red liquid when cut; grown on *Quercus* sp. (Figure 2a).

After isolation by aseptical explanting of hyphal tufts from freshly harvested fruiting bodies and subcultivation, *F. hepatica* mycelium was cultivated for four weeks at 25 °C on potato dextrose agar (PDA). Mycelial characteristics as recorded for *F. hepatica* strains by Stalpers and Vlug [39] on two different media, reappeared with our strain of *F. hepatica* on PDA: the mycelium displayed a whitish wooly to cottony texture of the mycelium that peripherally collapsed into a velutinous yellowishly colored mat (Figure 2b). The less intense yellow pigmentation in contrast to the cultures by Stalpers and Vlug [39] is in agreement with the work of Griffith et al. [40], even though the latter work only included ascomycetes. In further agreement with its identity, microscopy of *F. hepatica* mycelium of our strain on PDA yielded typical features of *F. hepatica* cultures described by Stalpers and Vlug [39]. Clamp connections were noticed on a regular basis on dikaryotic hyphae (Figure 2c). Some clamps grew out to form new hyphae (Figure 2d). Also, the typical branching of hyphae was noticed, which normally takes place either at acute angles (Figure 2d) or the branching-off of very narrow hyphae growing from wide hyphae could be observed (Figure 2c).

### 3.3. Identification of the Antifungal Polyyne Feldin from Basidiomycete Extract BE05

**Figure 3:**
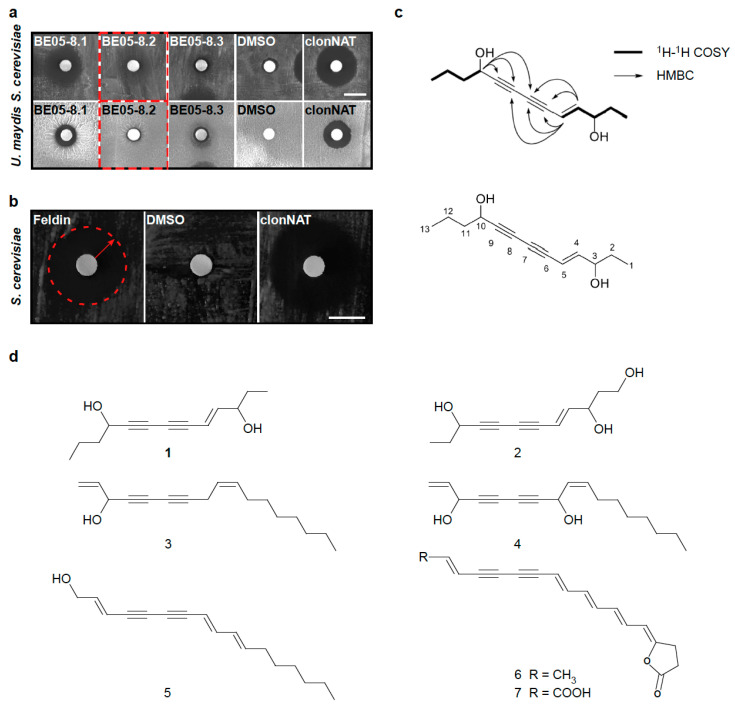
Bioactivity-guided fractionation from the basidiomycete ethyl acetate crude extract BE05; bioactivity of the freshly isolated bioactive compound against *S. cerevisiae*, and structure analysis of this compound (feldin). (**a**) Bioactivity test of fraction 8.2 (highlighted by a dashed frame) and two adjacent fractions from basidiomycete ethyl acetate crude extract BE05 against *S. cerevisiae* and *U. maydis*. The biotesting with fraction 8.2 yielded a zone of growth inhibition with *S. cerevisiae* (halo), while resulting in minimal growth inhibition with *U. maydis*. In total, 100 µg of this fraction were dissolved in DMSO to impregnate the filter paper disk. DMSO was used instead of the extract as negative control and 200 µg of clonNAT dissolved in ddH_2_O was used as positive control, respectively. The scale bar represents 1 cm. Three independent biological experiments (n = 3) were carried out. (**b**) Bioactivity test against *S. cerevisiae* of a subsample of the *F. hepatica* polyyne feldin, which was directly saved from fraction 8.2.6 before running NMR. The biotesting yielded a zone of growth inhibition (halo, dashed red circle with arrow) with *S. cerevisiae*. Here, 100 µg of feldin was dissolved in DMSO to impregnate the filter paper disk. DMSO was used instead of the extract as negative control and 200 µg of clonNAT dissolved in ddH_2_O was used as positive control, respectively. The scale bar represents 1 cm. Three independent biological experiments (n = 3) were carried out. (**c**) Structure of the *F. hepatica* polyyne feldin. (**d**) Similarity of chemical structure of feldin with known polyynes (1 in bold face, feldin; 2, 4-dodecene-6,8-diyne-1,3,10-triol; 3, falcarinol; 4, falcarindiol; 5, oenanthetol; 6, xerulin; 7, xerulinic acid).

According to the bioactivity results (see Figure 1), we largely upscaled the cultivation of *F. hepatica* on PDA, followed by ethyl acetate extraction and LC-MS-based fractionation and subfractionation of a large amount of BE05 (see Materials and Methods). All fractions were retested for their antifungal activity against *S. cerevisiae* and *U. maydis.* Fractions showing growth inhibition of *S. cerevisiae* but hardly of *U. maydis*, such as fraction 8.2, were further processed (Figure 3a). Applying LC-MS-mediated clean-up, we were able to purify one bioactive fraction (fraction 8.2.6) containing only one promising mass signal, which was structure-elucidated via NMR. To obviate potential substance instability that could result in substance decomposition after NMR—a known phenomenon with some natural products (see discussion)—we immediately saved a subsample of freshly obtained fraction 8.2.6. This subsample was immediately applied to biotesting against *S. cerevisiae* (Figure 3b), leaving just enough of the fraction 8.2.6 (2.0 mg) to execute NMR analysis to resolve the structure.

The molecular formula of the corresponding compound (Figure 3c), designated as feldin, was established from the quasimolecular [M + Na]^+^ ion peak at *m*/*z* 229.1178 (calcd for C_13_H_18_O_2_Na, 229.1199, ∆ppm 9.2), with a degree of unsaturation of five. All proton signals were associated with their respective carbons via analysis of the HSQC spectrum. The resemblance of the NMR data of feldin (Table 2) and 4-dodecene-6,8-diyne-1,3,10-triol [41], another polyyne, suggested that they were structurally similar, except that C-1 hydroxymethyl in 4-dodecene-6,8-diyne-1,3,10-triol is replaced by a methyl group (δ_C_ = 8.6) in feldin. These structural differences were determined by 2D NMR data (Figure S3–S7). In addition, feldin has one more methylene at C-12 (δ_C_ = 18.1) than 4-dodecene-6,8-diyne-1,3,10-triol. Furthermore, feldin also exhibits some structural resemblance to falcarinol and falcarindiol from carrot roots [25,26,27]; oenanthetol from leaves, as well as seeds, of *Trachyspermum ammi* (L.) Spr. [42]; and also with xerulin as well as xerulinic acid [43] from the fir-wood decomposing agaric *Xerula melanotricha* (Figure 3d). In parallel, employing the subsample of feldin saved before NMR, we ascertained bioactivity of this compound against *S. cerevisiae* (see Figure 3b). In essence, we, thus, not only succeeded in solving the structure of the compound feldin from the basidiomycete *F. hepatica*. We recorded that growth of the ascomycete *S. cerevisiae* is inhibited when it is exposed to feldin.

## 4. Discussion

In the present study, we have bioactivity-screened PDA culture-derived ethyl acteate extracts of 41 strains, representing 35 basidiomycete mushroom species. One out of the 41 strains produced an extract that displayed antifungal bioactivity. From this strain of *F. hepatica*, we characterized a bioactive compound via a bioactivity-guided isolation, employing the model ascomycete *S. cerevisiae* and the model basidiomycete *U. maydis* as test microorganisms. Pure bioactive compound isolation and structure analysis revealed the bioactive polyyne (‘polyacetylene’) named feldin.

Comparing the present study to the work by Suay et al. [44], who extensively screened Southern European mushroom strain methanolic extracts for potential antimicrobial bioactivities without isolating and analyzing any pure bioactive compounds, we observed a lower “hit rate” of extracts showing antimicrobial bioactivity. Our hit rate was only 2.4%, while the hit rate of those authors was 45.1%. However, their general hit rate for antifungal extracts was comparable to ours, i.e., 6% to our 2.4%. The detected discrepancy of the general hit rates may at least partially relate to the cultivation technique employed by Suay et al. [44], who cultivated in more nutritious aerated liquid medium (8% glucose, 5% corn meal), whereas the here-employed fungi were cultivated on PDA medium (2% glucose, 5.75% potatoes [40]). Accordingly, the average amount of crude extract solids and of different secondary metabolites per strain produced by at least some of their fungi may have, thus, generally been higher compared to the one obtained per strain in the present study. On the one hand, this implies that a more extensive screening of the strains in the present study (more diverse cultivation regimes including different liquid media and solid-state media) would certainly lead to a higher diversity of secondary metabolites and a higher bioactivity hit rate due to the OSMAC (“one strain many compounds”) effect [31,45,46]. Still, media that lead to increased fungal growth and large amounts of crude extract solids, or high metabolic diversity for one fungal strain, may induce poor metabolic diversity for a different fungal strain, or not serve well for production of certain compounds of interest within the same strain [47].

Although known from fungi, polyynes from plants are even better studied, such as falcarinol and falcarindiol from oil-filled channels within the periderm/pericyclic parenchyma tissue running parallel to the length of the root of carrot plants. Falcarindiol exhibits antifungal bioactivity protecting the young roots, supposedly via alteration or damage of the plasma membrane or other membrane functions [25,26,27], which is also a proposed mode of action of certain bacterial polyynes [48]. In addition, certain falcarindiol isoforms display antibacterial bioactivities [49,50]. Being potent antibacterials, some such polyynes have been the source of a number of unique pharmacophores, like phomallenic acid C identified from a *Phoma* sp. (Ascomycota). This one exhibits a 20-fold higher potency than thiolactomycin or cerulenin against the Gram-positive human-pathogenic bacterium *Staphylococcus aureus* [51].

So far, some polyynes from *F. hepatica* [19,21] have been described, as well as from *F. pallida,* including a polyyne glycoside [52]. The two structurally similar polyynes, classified as triacetylene derivatives, the Cinnatriacetins A and B, were isolated from *F. hepatica* basidiomes and reported to exhibit bioactivity against Gram-positive bacteria, but none against Gram-negative bacteria and *S. cerevisiae* [21]. This, and the fact that the polyyne feldin we describe originates from *F. hepatica* mycelium showing activity against *S. cerevisiae*, supports the notion that *F. hepatica* may employ a versatile arsenal of polyynes to equip its chemical defense system against antagonists, including fungal ones. Well-known with cultivated mushroom species like the button mushroom (*Agaricus bisporus*), mushroom-parasitic bacteria and microfungi impose a severe threat to mushrooms during substrate colonizing or basidiome formation. Blotch-disease-causing pseudomonads [53] or the ascomycetous button mushroom pathogen *Lecanicillium fungicola* [54] are certainly among the economically most relevant representatives of such mushroom parasites. Thus, in nature, polyynes accumulating in *F. hepatica* mycelium and fruiting bodies may potentially help the fungus to keep such antagonists at bay. In contrast to induced mushroom defense systems, such as the antifungal strobilurin A of *Oudemansiella mucida* [55], the polyyne feldin from *F. hepatica* we describe here belongs to the autonomous defense molecules [4], at least under the tested conditions, as it is constitutively produced by its mycelium on PDA. This is well in line with the fact that it is relatively common to observe the accumulation of such compounds in fungi, in contrast to the situation in plants [27].

Fungal polyynes, such as 10-hydroxy-undeca-2,4,6,8-tetraynamide, may display broad bioactivity spectra. Besides activity against Gram-negative and Gram-positive bacteria, it also affects a broad spectrum of fungi (several ascomycetes, including *S. cerevisiae,* as well as the closely related human-pathogenic yeast *C. albicans*, and the basidiomycetous yeast *Rhodotorula glutinis*), the oomycete *Phytophtora infestans*, and Ehrlich ascites tumor cells [56]. Other fungal polyynes, like the allenic fungal polyyne of Schlingmann et al. [14], show a narrower bioactivity spectrum. While basidiomycetes like *U. maydis* and *Rhodotorula rubra* are hardly affected, it is strongly active against Gram-positive bacteria and *C. albicans*.

Fungal polyynes have been studied as potential drugs of high potency, like the tuberculosis cure mycomycin [57], the cholesterol biosynthesis inhibitors from the silver fir wood-decomposer *X. melanotricha* [43], or 10-hydroxy-undeca-2,4,6,8-tetraynamide from *Mycena viridimarginata* [56]. In this context, stability and cytotoxicity are major challenges to the applicability of the individual substance under study [43,56,57]. Polyynes tend to be unstable succumbing, for example, to oxidative, photolytic, and/or pH-dependent decomposition [27]. Trying to obviate that the bioactive compound in subfraction 8.2.6 (which was revealed as a polyyne after NMR) might get damaged after NMR solvent evaporation, as is known with unstable natural products like polyynes [48,58,59], bioactivity of feldin against *S. cerevisiae* was ascertained with a subsample of fresh feldin saved before NMR (see Figure 3b). Similarly, the antibacterial polyyne isolated from the basidiomycete *Baeospora myosura* proved to be very unstable, i.e., it polymerized when the solvent was removed [58]. Likewise, the 3,4,5,6-tetrahydro-6-hydroxy-derivative of the tuberculosis cure mycomycin (reported as potent but unstable by Celmer and Solomons [57]) was reported to be too unstable to isolate in a pure form [14]. Moreover, bacterial polyynes can also be unstable for the same or similar reasons. This makes very careful handling indispensable for bioactivity assessment, e.g., by avoiding light, oxygen, and solvent evaporation as much as possible [48,59]. Feldin apparently got damaged only after NMR solvent evaporation. Potentially, as in the case of the antibacterial polyyne from the basidiomycete *B. myosura* [58], polymerization has happened causing a loss of bioactivity. Accordingly, we cautiously assume that a customized minimalistic NMR solvent evaporation would be required to avoid feldin disintegration after NMR.

A possible solution for stabilizing bioactive polyynes like feldin may come from a (temporary) derivatization, e.g., creating a polyyne glycoside. Such derivatives may be found in fungi and plants and can exhibit antibacterial and anti-inflammatory activities [60,61,62]. Even in a very close relative of *F. hepatica* a natural polyyne glycoside is known [52]. Depicting a conjugation of a sugar moiety and a polyyne, such glycosides are judged as more stable than pure polyynes. Interestingly, no bioactivity was reported in the one described by Ahmed et al. [52]. This may relate to one suggested mode of action of polyynes. Bäuerle et al. [56] discussed the activity of 10-hydroxy-undeca-2,4,6,8-tetraynamide as correlated to its high chemical reactivity due to carbon–carbon triple bonds. Accordingly, such compounds, as stated by Walsh et al. [63], cause inactivation of enzymes by alkylation. Pan et al. [60] suggested that the carbohydrate part of polyyne glycosides might play a ‘protecting’ role in either stabilizing the polyyne moiety or in masking its biological activity until cleavage of the carbohydrate moiety by a glycosidase. Otherwise, it may increase solubility and facilitate the delivery of these molecules to certain cell types, e.g., via binding to certain sugar transporters.

## 5. Conclusions

In this exemplary study, we characterized the unstable bioactive mushroom polyyne feldin which at least qualitatively appears to be more toxic to the model ascomycete *S. cerevisiae* than to the basidiomycete model fungus *U. maydis*. This can be used as a starting point for future studies examining whether basidiomycetes like *U. maydis* may generally be more tolerant to prominent basidiomycete-derived eukaryotic toxic compounds, such as stobilurins, illudins, or even basidiome-derived antifungals like ageritin [2,5,6,64]. Such may be achieved by exposing *U. maydis* to a selection of these compounds. Tests may also include yet-to-discover differentially bioactive compounds from basidiomycetes by applying a more extensive screening. The latter should include additional strains also of the same species [28,65], a few more test microorganisms, and additional cultivation setups that are known to elicit the OSMAC effect [31,45,46], such as liquid media or bulk solid-state media. If this revealed a higher tolerance of *U. maydis* against certain such compounds, consequently *U. maydis* might be considered a candidate host for heterologous expression of mushroom-derived biologicals.

Moreover, if an understanding of polyyne biosynthesis were desirable, heterologous expression, including a derivatization approach, could be meaningful. Following identification of potential polyyne biosynthesis genes in the *F. hepatica* genome sequence of Floudas et al. [16], overexpression of respective candidate genes should be attempted, similar to the proof-of-principle approach recently accomplished by Lee et al. [66] on sesquiterpene production. Simultaneous expression of plant enzymes for a coupling approach might, eventually, allow the engineering of polyyne-derivatives, which are relatively stable and secreted into the culture medium. In a final step, the conjugation could be cleaved by a glycosidase to release the engineered compound’s polyyne moiety.

## Figures and Tables

**Table 1:** Forty-one mushroom strain extracts (BE01-41) tested for potential antifungal bioactivity.

Extract	Species	Strain	Geographic ^1^ and Ecological Data
BE01	*Armillaria ostoyae*	P089	Göttingen (Gö), Kerstlingeröder Feld, under *Pinus nigra*, tc ^2^, leg., det. M.G. (2009-10-07)
BE02	*Armillaria* cf. *ostoyae*	P145	1.2 km S of Grobsdorf (Gd), Nordhalde (Nh), lc ^3^, *Tilia* sp. stump, leg., det. M.G. (2010-09-27)
BE03	*Coprinopsis atramentaria*	P125	Jena (J), Beutenberg, half buried softwood, lc, leg., det. M.G. (2013-11-18)
BE04	*Coprinopsis picacea*	P106	1 km W of Gö-Herberhausen (GöHe), *Fagus sylvatica* forest (Fsf), tc, leg., det. M.G. (2013-11-25)
BE05	*Fistulina hepatica*	P052	J, 1 km SW of “Steinkreuz Ziegenhain”, Fsf, lc, *Quercus* sp. stump, leg., det. F.H. (2013-08-18)
BE06	*Flammulina velutipes*	P111	J, Cospedaer Grund, lc, *Fraxinus excelsior* stump, leg., det. M.G. (2009-12-01)
BE07	*Fomes fomentarius*	P116	Jonsdorf, Weißer Stein, lc, *Betula pendula* wood, leg., det. M.G. (2012-03-05)
BE08	*Pleurotus dryinus*	P090	Dederstedt, lc, living stem of *F. excelsior*, leg., det. Dr. habil. H. Dörfelt (2009-11-18)
BE09	*Pleurotus* cf. *pulmonarius*	P046	Oberursel-Hohemark (OHm), mixed Fsf, lc, *F. sylvatica* wood, leg., det. F.H. (2013-09-13)
BE10	*Pleurotus ostreatus*	P118	Hannoversche Klippen 1.5 km NW of Bad Karlshafen, lc, *F. sylvatica* wood, leg., det. M.G. (2012-07-30)
BE11	*Agaricus arvensis*	P151	Kauern, Nh, 1 km S of Gd, mixed forest, tc ^2^, leg., det. M. G. (2009-09-23)
BE12	*Agaricus augustus*	P096	2.5 km NE of Maria Laach (ML), mixed Fsf, tc, leg., det. M. G. (2010-08-12)
BE13	*Agaricus augustus*	P148	Gö., Brüder-Grimm-Allee, tc, under *Tilia* sp., leg., det. M. G. (2013-10-25)
BE14	*Auricularia auricula-judae*	P093	J, Mühltal, lc ^3^, *Sambucus nigra* wood, leg., det. M. G. (2009-12-07)
BE15	*Bovista nigrescens*	P123	Rothesütte, about 500 m SE of Rothesütte, tc, pasture, leg., det. M. G. (2013-09-23)
BE16	*Clitocybe geotropa*	P149	Gö, close to GöHe, Fsf, tc, leg., det. M. G. (2013-12-02)
BE17	*Clitocybe odora*	P053	Morgenröthe, mixed *Picea abies* forest, tc, needle litter, leg., det. F. H. (2013-09-07)
BE18	*Galerina marginata*	P057	OHm, mixed Fsf, lc, *P. abies* wood, leg., det. F. H. (2013-09-13)
BE19	*Ganoderma lucidum*	P095	Königsfeld/SW, 500 m N of Königsfeld, lc, *Quercus* sp. stump, leg., det. M. G. (2010-07-17)
BE20	*Gloeophyllum odoratum*	P124	J, Mühltal, lc, *P. abies* stump, leg., det. M. G. (2013-11-04)
BE21	*Hypholoma fasciculare*	P099	750 m SE of Gd, mixed forest, lc, *Quercus* sp. stump, leg., det. M. G. (2010-09-07)
BE22	*Hypholoma sublateritium*	P169	200 m S of Closewitz, *Quercus* spp. forest, lc, *Quercus* sp. stump, leg., det. M. G. (2014-02-17)
BE23	*Inonotus obliquus*	P150	Oybin, Töpfer/Brandhöhe, lc, *B. pendula* wood, leg., det. M. G. (2014-01-06)
BE24	*Kuehneromyces mutabilis*	P050	OHm, mixed Fsf, lc, on *F. sylvatica* wood, leg., det. F. H. (2013-09-13)
BE25	*Langermannia gigantea*	P051	Bad Berka, Trebestrasse, tc, garden lawn, leg., det. F. H. (2013-08-17)
BE26	*Lycoperdon excipuliforme*	P122	800 m S of Gd, tc, under scattered *B. pendula* trees, leg., det. M. G. (2013-09-12)
BE27	*Lycoperdon molle*	P154	ML, Laacher-See-Haus, 1.3 km SE of ML, on a path in a Fsf, tc, leg., det. M. G. (2010-08-12)
BE28	*Macrolepiota procera*	P097	ML, 2.5 km NE of ML, mixed Fsf, tc, leg., det. M. G. (2010-08-16)
BE29	*Macrolepiota procera*	P130	Kauern, Nh, 1.2 km S of Gd, mixed forest, tc, leg., det. M. G. (2009-09-25)
BE30	*Marasmius oreades*	P144	1 km S of Gd, tc, grassy forest margin, leg., det. M. G. (2010-09-08)
BE31	*Marasmius scorodonius*	P055	OHm, mixed Fsf, tc, *F. sylvatica* leaf litter, leg., det. F. H. (2013-09-13)
BE32	*Megacollybia platyphylla*	P026	2.5 km N of Rambach, 2 km NW of Naurod, mixed Fsf, tc, leg., det. F. H. (2013-05-27)
BE33	*Pleurotus ostreatus*	P114	ML, Laacher-See-Haus, 1.3 km SE of ML, lc, on *F. sylvatica* wood, leg., det. M. G. (2011-08-02)
BE34	*Pleurotus ostreatus*	P119	Rosdorf, Kiessee, lc, on wood of *Salix* sp., leg., det. M. G. (2013-08-23)
BE35	*Pleurotus ostreatus*	P128	Hainewalde, near graveyard, lc, *Populus* x *canadensis* stump, leg., det. M. G. (2014-01-06)
BE36	*Polyporus brumalis*	P117	1.5 km SW of GöHe, mixed Fsf, lc, *Tilia* sp. wood, leg., det. M. G. (2012-03-05)
BE37	*Polyporus tuberaster*	P036	J, 300 m S of “Steinkreuz Ziegenhain”, Fsf, lc, on decayed wood, leg., det. F. H. (2013-08-18)
BE38	*Sarcomyxa serotina*	P126	800 m W of GöHe, mixed Fsf, lc, *F. sylvatica* stump, leg., det. M. G. (2013-11-25)
BE39	*Stropharia aeruginosa*	P102	Jenaprießnitz, Tännicht, Fsf, tc, leg., det. M. G. (2010-10-06)
BE40	*Trametes gibbosa*	P129	J, Mühltal, 1.3 km SW of Cospeda, *F, sylvatica* wood, leg., det. M. G. (2013-03-17)
BE41	*Tricholomopsis rutilans*	P056	OHm, mixed Fsf, lc, *F. sylvatica* wood, leg., det. F. H. (2013-09-13)

^1^ Fungal material was collected in Germany; ^2^ terricolous; ^3^ lignicolous.

**Table 2:** NMR data assignment of feldin.

No.	^1^H (mult., *J*)	^13^C, mult.
1	0.95 (t, 6.8)	8.6, CH_3_
2	1.53 (m)	29.3, CH_2_
3	4.06 (td, 7.1, 1.5)	72.4, CH
4	6.32 (15.9, 5.6)	149.8, CH
5	5.78 (ddd, 15.9, 1.7, 0.7)	107.3, CH
6	-	76.0, C
7	-	72.8, C
8	-	68.0, C
9	-	83.2, C
10	4.41 (t, 6.7)	61.5, CH
11	1.68 (m)	39.5, CH_2_
12	1.48 (m)	18.1, CH_2_
13	0.98 (t, 6.7)	12.6, CH_3_

## Supplementary Materials

The following are available online at https://www.mdpi.com/2218-273X/10/11/1502/s1, Figure S1: Bioactivity test of basidiomycete crude extracts against *S. cerevisiae* (a) and *U. maydis* (b), Figure S2: Bioactivity test with crude extract BE05 against *C. glutamicum* and *E. coli*, Figure S3: ^1^H NMR spectrum of feldin in MeOH-*d*_4_, Figure S4: ^13^C NMR spectrum of feldin in MeOH-*d*_4_, Figure S5: HSQC spectrum of feldin in MeOH-*d*_4_, Figure S6: HMBC spectrum of feldin in MeOH-*d*_4_, Figure S7: ^1^H-^1^H COSY spectrum of feldin in MeOH-*d*_4_.
