# Supplementary material for: Identification of Feldin, an Antifungal Polyyne from the Beefsteak Fungus Fistulina hepatica
Source: Biomolecules. 2020 Oct 31;10(11):1502. doi: 10.3390/biom10111502 (PMC7692509; doi:10.3390/biom10111502)
Supplement: Supplementary file 1 [file biomolecules-10-01502-s001.pdf]

biomolecules

MDPI

Article

Identification of Feldin, an Antifungal Polyyne from the Beefsteak Fungus *Fistulina hepatica*

Jungho Lee [1], Yi-Ming Shi [2], Peter Grün [2], Matthias Gube [3], Michael Feldbrügge [1], Helge Bode [2,4,5] and Florian Hennicke [6,*]

[1] Institute for Microbiology, Cluster of Excellence on Plant Sciences, Bioeconomy Science Centre, Heinrich Heine University Düsseldorf, 40204 Düsseldorf, Germany; jungho.lee@hhu.de (J.L.); feldbrue@hhu.de (M.F.)
[2] Molecular Biotechnology, Department of Biosciences, Goethe University Frankfurt, 60438 Frankfurt am Main, Germany; Shi@bio.uni-frankfurt.de (Y.-M.S.); p.gruen@bio.uni-frankfurt.de (P.G.); h.bode@bio.uni-frankfurt.de (H.B.)
[3] Soil Science of Temperate Ecosystems, Georg-August University Göttingen, 37077 Göttingen, Germany, mgube@uni-goettingen.de
[4] Buchmann Institute for Life Sciences (BMLS), Goethe University Frankfurt, 60438 Frankfurt am Main, Germany
[5] Senckenberg Gesellschaft für Naturforschung, 60325 Frankfurt, Germany
[6] Project Group Genetics and Genomics of Fungi, Chair Evolution of Plants and Fungi, Ruhr-University Bochum (RUB), Universitätsstr. 150, 44780 Bochum, Germany
* Correspondence: florian.hennicke@ruhr-uni-bochum.de

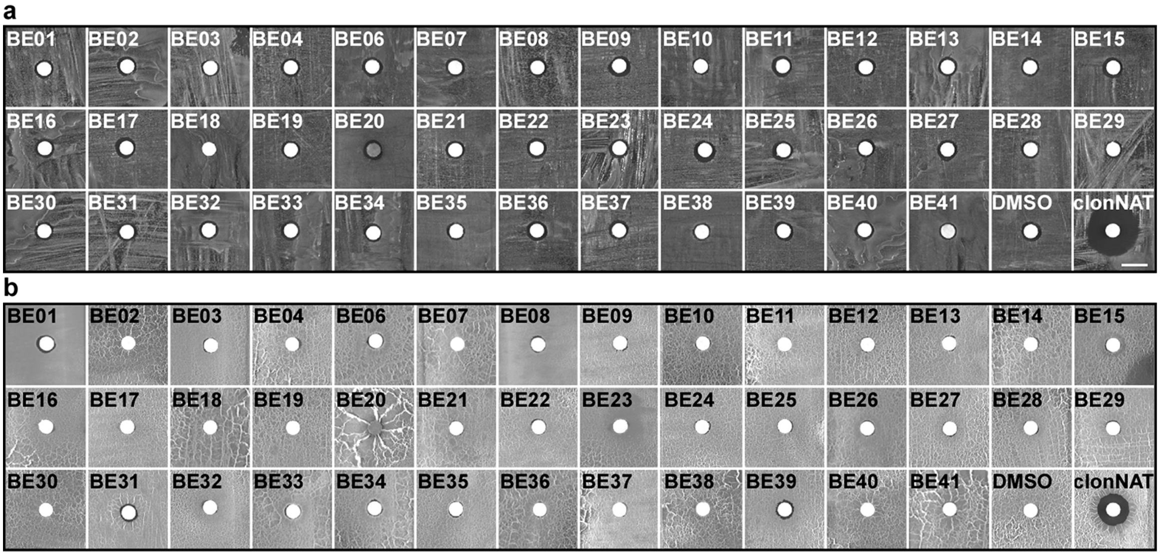

Figure S1. Bioactivity testing of forty ethyl acetate crude extracts from forty basidiomycete strains against *Saccharomyces cerevisiae* (**a**) and *Ustilago maydis* (**b**). 100 µg of each basidiomycete culture crude extract (BE01-BE04, BE06-BE41) was dissolved in DMSO to impregnate the filter paper disk. DMSO was used instead of such an extract as negative control and 200 µg of clonNAT dissolved in ddH_2O was used as the positive control, respectively. The scale bar represents 1 cm. Three independent biological experiments (n=3) were carried out.

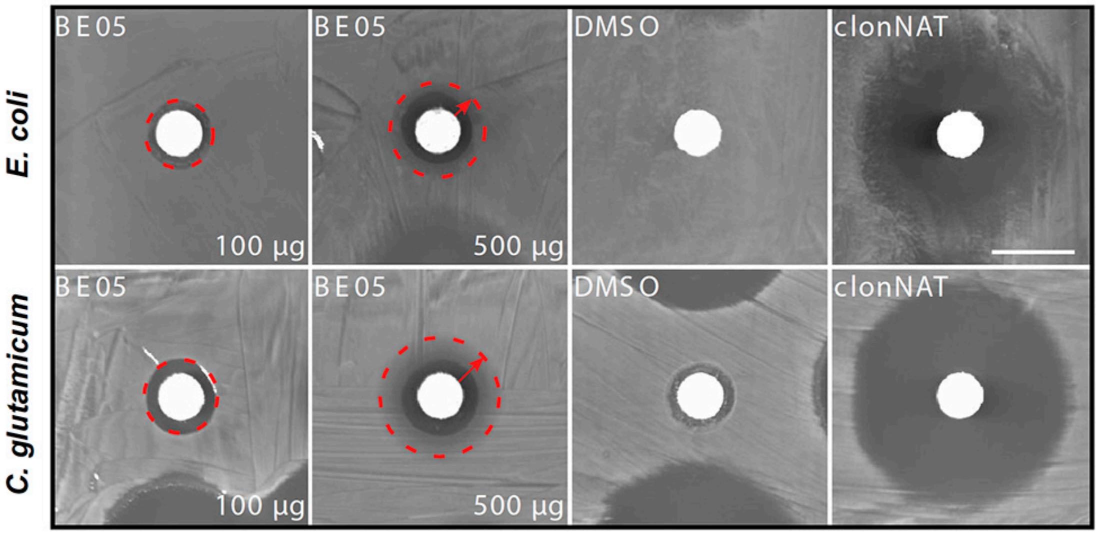

Figure S2. Bioactivity testing of the ethyl acetate crude extract BE05 against *Corynebacterium glutamicum* and *Escherichia coli*. 100 µg or 500 µg of BE05 were dissolved in DMSO to impregnate the filter paper disk. DMSO was used instead of such an extract as negative control and 200 µg of clonNAT dissolved in ddH_2O was used as the positive control, respectively. The dashed red circle and arrow indicate the size of the growth inhibition zone (halo) with both bacterial strains caused by 500 µg of BE05. This halo comprises an inner zone clearly delimited by its dark color from the agar medium below (no bacterial biofilm), and an outer zone (still some bacterial biofilm) which appears brighter than the inner halo but darker than the surrounding bacterial biofilm outside the dashed circle. The scale bar represents 1 cm. Three independent biological experiments (n=3) were carried out.

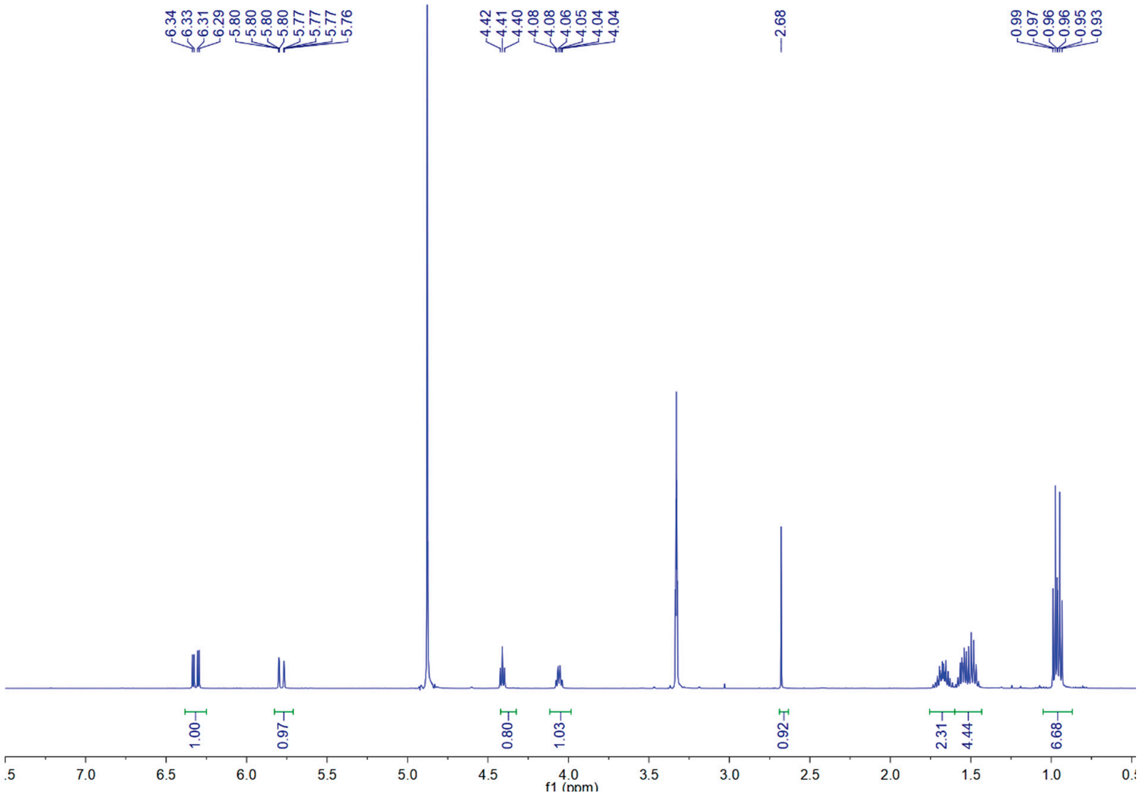

Figure S3. ^1H NMR spectrum of feldin in MeOH-d_4.

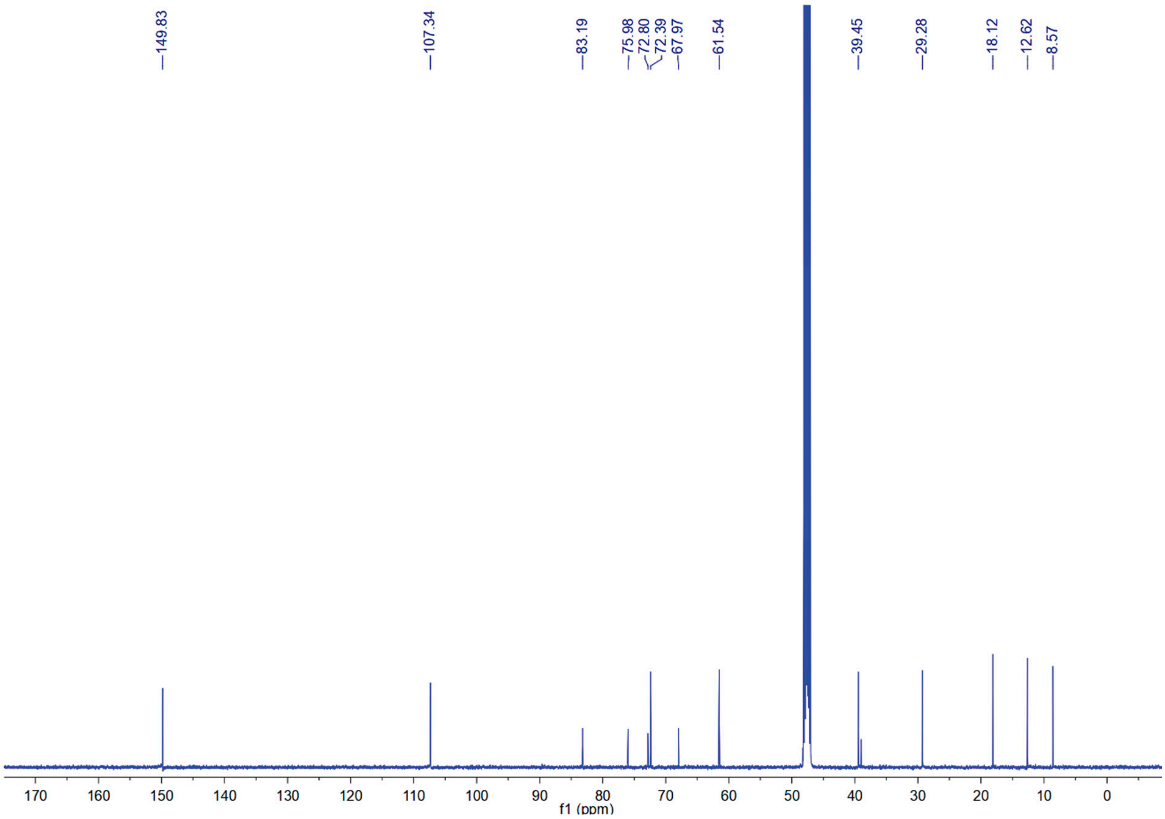

Figure S4. ^{13}C NMR spectrum of feldin in MeOH-d_4.

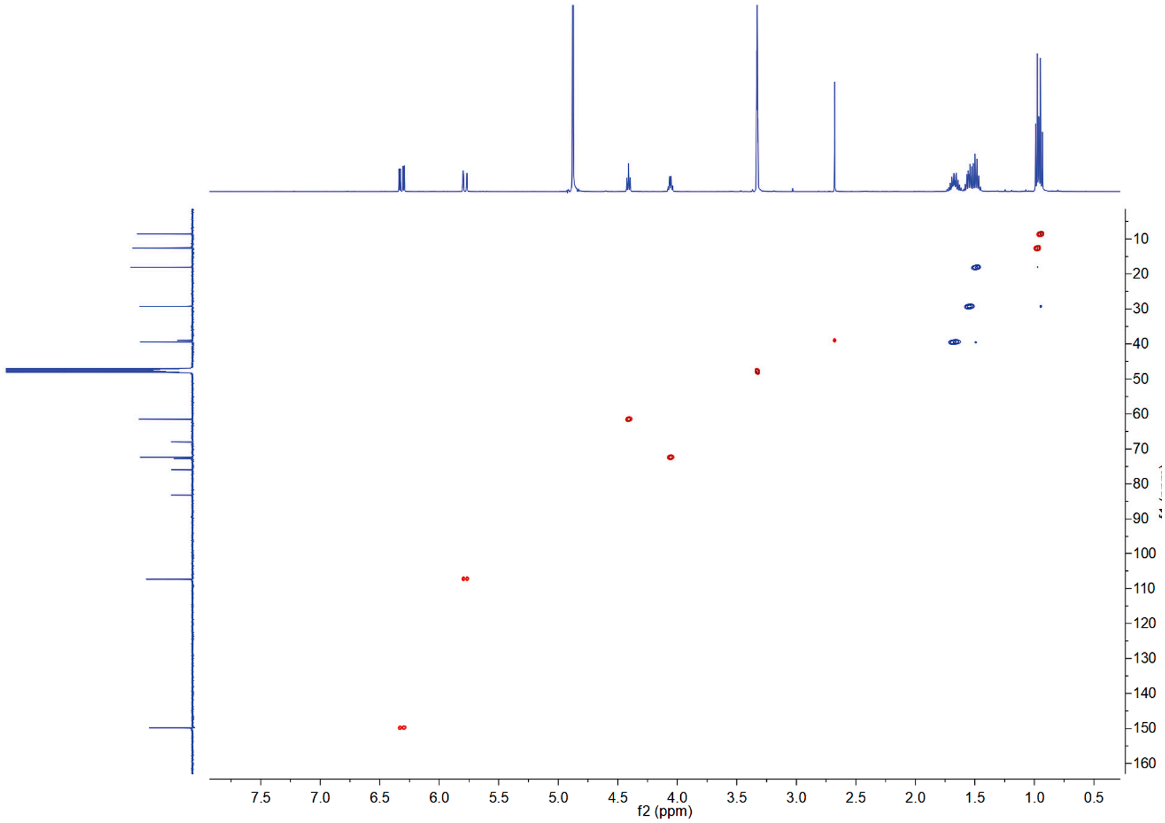

Figure S5. HSQC spectrum of feldin in MeOH-d_4.

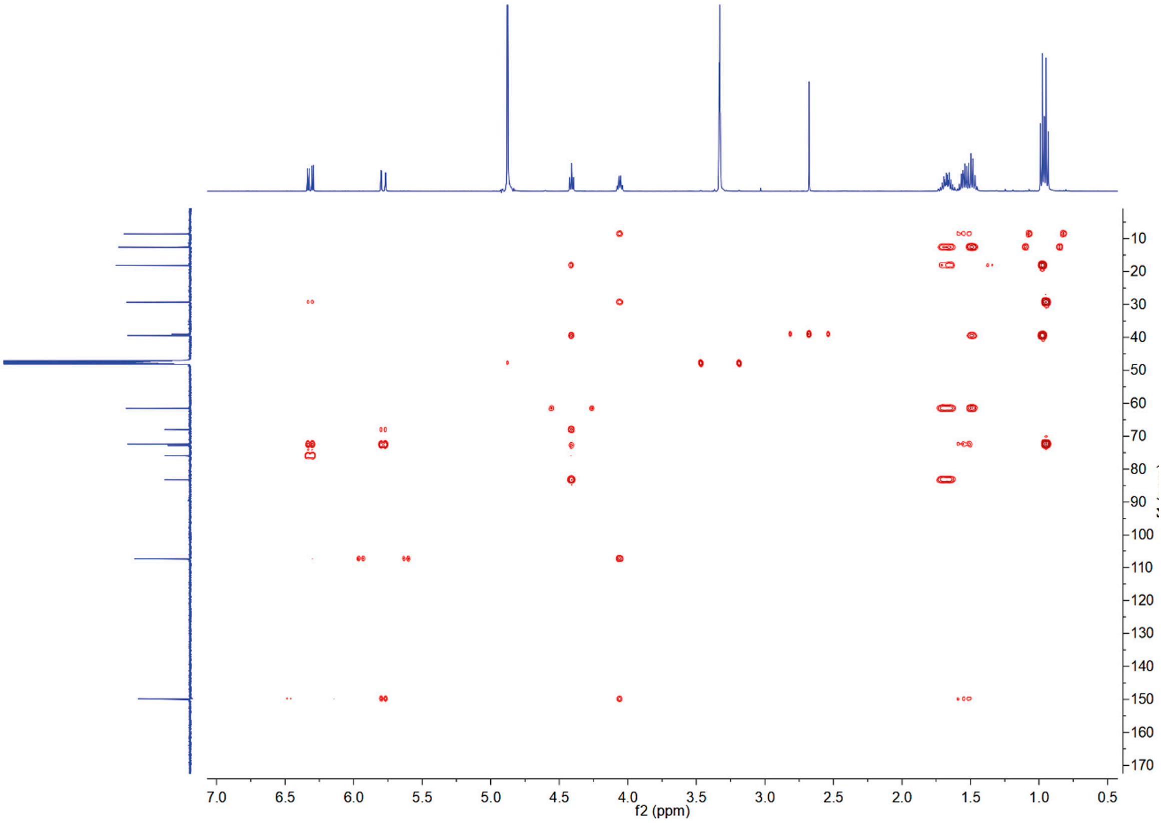

Figure S6. HMBC spectrum of feldin in MeOH-d_4.

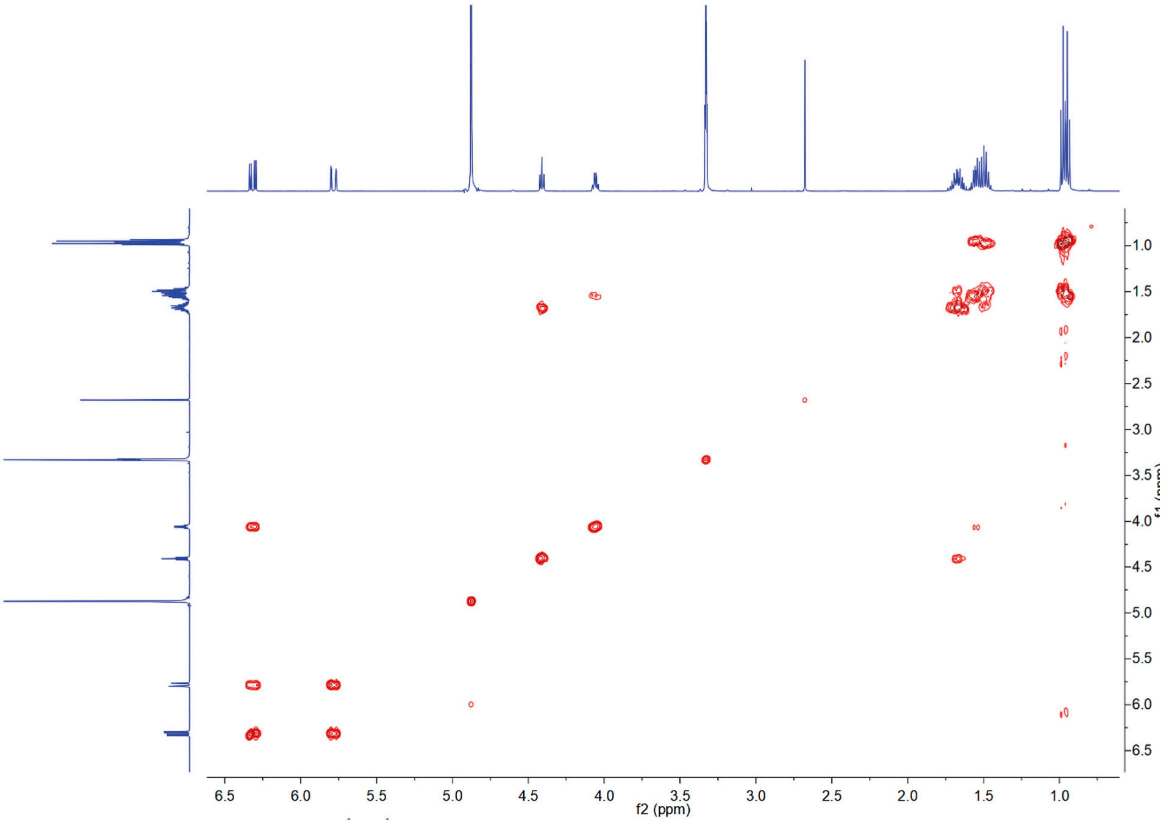

Figure S7. ^{1}H-^{1}H COSY spectrum of feldin in MeOH-d_4.

© 2020 by the authors. Licensee MDPI, Basel, Switzerland. This article is an open access article distributed under the terms and conditions of the Creative Commons Attribution (CC BY) license (http://creativecommons.org/licenses/by/4.0/).
